# Weakly supervised learning of RNA modifications from low-resolution epitranscriptome data

**DOI:** 10.1093/bioinformatics/btab278

**Published:** 2021-07-12

**Authors:** Daiyun Huang, Bowen Song, Jingjue Wei, Jionglong Su, Frans Coenen, Jia Meng

**Affiliations:** Department of Biological Sciences, Xi’an Jiaotong-Liverpool University, Suzhou, Jiangsu 215123, China; Department of Computer Science, University of Liverpool, Liverpool L69 7ZB, UK; Department of Mathematical Sciences, Xi’an Jiaotong-Liverpool University, Suzhou, Jiangsu 215123, China; Institute of Systems, Molecular and Integrative Biology, University of Liverpool, Liverpool L69 7ZB, UK; Department of Mathematical Sciences, Xi’an Jiaotong-Liverpool University, Suzhou, Jiangsu 215123, China; School of AI and Advanced Computing, XJTLU Entrepreneur College (Taicang), Xi’an Jiaotong-Liverpool University, Suzhou, Jiangsu 215123, China; AI University Research Centre, Xi’an Jiaotong-Liverpool University, Suzhou, Jiangsu 215123, China; Department of Computer Science, University of Liverpool, Liverpool L69 7ZB, UK; Department of Biological Sciences, Xi’an Jiaotong-Liverpool University, Suzhou, Jiangsu 215123, China; Institute of Systems, Molecular and Integrative Biology, University of Liverpool, Liverpool L69 7ZB, UK; AI University Research Centre, Xi’an Jiaotong-Liverpool University, Suzhou, Jiangsu 215123, China

## Abstract

**Motivation:**

Increasing evidence suggests that post-transcriptional ribonucleic acid (RNA) modifications regulate essential biomolecular functions and are related to the pathogenesis of various diseases. Precise identification of RNA modification sites is essential for understanding the regulatory mechanisms of RNAs. To date, many computational approaches for predicting RNA modifications have been developed, most of which were based on strong supervision enabled by base-resolution epitranscriptome data. However, high-resolution data may not be available.

**Results:**

We propose WeakRM, the first weakly supervised learning framework for predicting RNA modifications from low-resolution epitranscriptome datasets, such as those generated from acRIP-seq and hMeRIP-seq. Evaluations on three independent datasets (corresponding to three different RNA modification types and their respective sequencing technologies) demonstrated the effectiveness of our approach in predicting RNA modifications from low-resolution data. WeakRM outperformed state-of-the-art multi-instance learning methods for genomic sequences, such as WSCNN, which was originally designed for transcription factor binding site prediction. Additionally, our approach captured motifs that are consistent with existing knowledge, and visualization of the predicted modification-containing regions unveiled the potentials of detecting RNA modifications with improved resolution.

**Availability implementation:**

The source code for the WeakRM algorithm, along with the datasets used, are freely accessible at: https://github.com/daiyun02211/WeakRM

**Supplementary information:**

Supplementary data are available at *Bioinformatics* online.

## 1 Introduction

Increasing evidence suggests that post-transcriptional ribonucleic acid (RNA) modifications regulate essential biological processes and are related to the pathogenesis of various diseases including multiple cancers (Esteve-Puig *et al.*, 2020; Shulman and Stern-Ginossar, 2020; Zaccara *et al.*, 2019). Precise identification of RNA modification sites is essential for an in-depth understanding of the regulatory circuitry of RNA life. Over 170 distinct RNA modifications have been identified in living organisms to date (Boccaletto *et al.*, 2018), among which, more than 10 modifications have been shown to widely occur in the human transcriptome and can be profiled with high-throughput sequencing approaches (Li *et al.*, 2017; McCown *et al.*, 2020). Since wet experiments for studying the epitranscriptomes are usually laborious and expensive (Jones *et al.*, 2020), computational approaches have become increasingly popular as a useful alternative, especially for preliminary studies.

To date, many *in silico* methods have been developed for the computational prediction of RNA modification sites from RNA (or DNA) sequences as well as other predictive genomic features. Among them, SRAMP is one of the earliest and widely applied predictive approaches for m^6^A RNA methylation based on the Random Forests method from RNA sequences (Zhou *et al.*, 2016). Recently, by taking advantage of both sequence and 35 additional genomic features, the WHISTLE method has achieved the best performance in m^6^A site prediction to date (Chen *et al.*, 2019). Gene2Vec is a very powerful deep learning framework that supports m^6^A predictions, which are enhanced by employing word embeddings to represent RNA sequences (Zou *et al.*, 2019). Some recent works further developed computational algorithms to predict modifications from direct RNA sequencing data like Oxford Nanopore Technologies (Jenjaroenpun *et al.*, 2020; Liu *et al.*, 2019). Together, these effects have greatly improved our understanding of the localization and working mechanisms of various RNA modifications under different biological contexts; see for example the comprehensive recent reviews (Anreiter *et al.*, 2021; Chen *et al.*, 2020; Liu *et al.*, 2020).

A major limitation of epitranscriptome prediction approaches is that, to the best knowledge of the authors, all of them are based on strong supervision. Strong supervision-based approaches perform well on modifications with base (or high)-resolution data, but usually overlook the weakly supervised information of RNA sequences when applied to low-resolution datasets, such as 5-hydroxymethylcytidine (hm^5^C) and N4-acetylcytidine (ac^4^C). These two modifications can be detected by enrichment-based sequencing approaches, such as hMeRIP-seq and acRIP-seq, respectively (Arango *et al.*, 2018; Delatte *et al.*, 2016), from which we can identify the RNA modification-containing regions (or peaks enriched with signals of RNA modification) of around 100 nt resolution. As there usually exist multiple Cs within such regions, it is not exactly clear which one is the true modifiable nucleotide and which are non-modifiable ones. Although it is possible to further enhance the resolution by searching for the motif of a specific modification, our previous study showed that this remedy will generate a large number of false-positive sites due to random occurring sequence motifs located close to real modification sites (Chen *et al.*, 2019). Meanwhile, it is clear that predictive methods based on this remedy have very limited performance (Liu *et al.*, 2020) or very narrow applicable scope (Zhao *et al.*, 2019). To address the challenges of learning from low-resolution epitranscriptome data, we consider here a weakly supervised learning framework.

Weakly supervised learning is aimed at constructing predictive models by learning from weakly labeled data (Zhou, 2018). An important scenario is when there are only coarse-grained labels provided (or with only labels for bags but not for instances), for example, in the case of image analysis, when the labels are only available at image level but not at object level. In genomics, weakly supervised learning, especially multi-instance learning (MIL), has been intensively applied for studying protein–DNA interaction (Gao and Ruan, 2015, 2017; Zhang *et al.*, 2019, 2020), with the basic assumption that the sequences captured by CLIP (or ChIP-seq) technologies contain both the interacting and non-interacting elements with the proteins. We know only the label of the entire sequence, but it is not exactly clear which part of the sequence plays the key role, and a significant proportion of it may not contribute to the binding between DNA and protein at all. MIL3D (Gao and Ruan, 2015) first treated each probe sequence as a labeled bag, utilized decision trees and probabilities averaging methods to predict bag-level classes. MIL-TeamD (Gao and Ruan, 2017) extended MIL3D by using TeamD (Annala *et al.*, 2011) as the instance classifier. WSCNN and its updated version WSCNNLSTM (Zhang *et al.*, 2019, 2020) further applied convolution neural network (CNN) and long short-term memory (LSTM) to capture sequence features through learning. Additionally, weakly supervised learning (MIL) has also been used for the functional prediction of proteins (Wu *et al.*, 2014), protein splicing variants (Panwar *et al.*, 2016), microRNA target prediction (Bandyopadhyay *et al.*, 2015) and protein–protein interaction (Mei and Zhu, 2014). Conceivably, as low-resolution epitranscriptome data provided labels only at region level but not at single-nucleotide level, the problem of learning from it can be suitably formulated with the weakly supervised learning framework.

We propose WeakRM, a general weakly supervised learning framework for predicting RNA modifications from low-resolution epitranscriptome datasets, such as those generated from acRIP-seq or hMeRIP-seq. Our model takes labels at the sequence level (rather than a nucleotide level) as input and predicts the sub-regions that are most likely to contain the modification of interest. To the best of our knowledge, this is the first time that RNA modification prediction was formulated under the framework of weakly supervised learning. Additionally, compared to existing MIL algorithms, which were originally developed for transcription factor binding site (TFBS) prediction, our model achieved better performance in RNA modification site prediction with major improvements, i.e. using the gated attention (Ilse *et al.*, 2018) for result merging and using random cropping data augmentation. Attention-based MIL was first proposed for image analysis, allowing the model to assign learnable weights to each instance. This method can aggregate information from all instances while adapting to sparse site distribution and high correlation between instances. In addition, such weights also indicate the region of interest by selecting high-weight instances. Random cropping, from another perspective, takes advantage of the key feature of the related biotechnology and uses the natural divisibility of RNA modification peaks to improve the model performance. By randomly cutting the ‘bag’ to generate new inputs, our network model can see more cases and learn the patterns more effectively and robustly.

Evaluations on three independent datasets (corresponding to three different RNA modification types and their respective sequencing technologies) demonstrated the general effectiveness of our approach in predicting RNA modifications from low-resolution data. Our approach outperformed state-of-the-art MIL algorithms for genomic sequences, such as WSCNN, which was originally designed for TFBS prediction. Our approach captured motifs that are consistent with existing knowledge. Visualization of the predicted modification-containing regions unveiled the potentials of detecting RNA modifications with improved resolution. WeakRM should make a powerful and useful tool for learning RNA modifications with only low-resolution epitranscriptome data.

## 2 Materials and methods

### 2.1 Epitranscriptome data

The proposed WeakRM framework described below was tested on three independent epitranscriptome datasets of low-resolution (around 100 nt), which corresponded to three distinct RNA modifications (ac^4^C, hm^5^C and m^7^G) and their respective sequencing technologies (acRIP-seq, hMeRIP-seq and m^7^G-MeRIP-seq) (see Table 1). All three technologies are based on the FRIP-seq protocol described previously (Meng *et al.*, 2013), in which, the fragmented RNAs are immunoprecipitated by the antibody targeting the modifications of interests, and then the RNAs were purified for next generation sequencing. The reads were aligned to the reference genome, and peak calling was conducted to capture the regions enriched with signals of RNA modification (or the ‘peak’s) with around 100 nt resolution (Dominissini *et al.*, 2013; Meng *et al.*, 2013). The peak regions should contain the RNA modification signal, and are considered as ‘positive’. Meanwhile, only the non-peak regions of peak-carrying genes were used as the ‘negative’ regions to exclude false negatives due to condition-specific gene expression. The obtained ‘negative’ regions were randomly cropped to balance the length and number between regions. Due to limited sensitivity, the RNA modification sites located on very lowly expressed genes will be missing from epitranscriptome data. The genomic sequences within the positive and negative regions were then extracted from the whole genome assembly and then used in this study.

**Table 1.** Epitranscriptome data

Modification	Technology	Resolution	Sample size	Cell line	Species	GEO	Source
		(nt)	(positive versus negative)				
ac^4^C	acRIP-seq	∼100	8630 versus 11 912	HeLa	Homo sapians	GSE102113	Arango *et al*. (2018)
ac^4^C	acRIP-seq	∼100	21 542 versus 27 590	HeLa	Homo sapians	GSE102113	Arango *et al*. (2018)
hm^5^C	hMeRIP-seq	∼100	2347 versus 3557	S2	Drosophila	—	Delatte *et al*. (2016)
m^7^G	m^7^G-MeRIP-seq	∼100	6022 versus 9096	HeLa	Homo sapians	GSE112276	Zhang *et al*. 92019)
m^7^G	m^7^G-MeRIP-seq	∼100	6873 versus 10 230	HepG2	Homo sapians	GSE112276	Zhang *et al*. (2019)
m^7^G	m^7^G-seq	1	6032	HeLa	Homo sapians	GSE112276	Zhang *et al*. (2019)
m^7^G	m^7^G-seq	1	3333	HepG2	Homo sapians	GSE112276	Zhang *et al*. (2019)

a Base-resolution m7G-seq sites were used to verify the locating ability of WeakRM. The sample size does not include data augmentation.

Additionally, to further validate the trained WeakRM model, we also extracted the precise locations of m^7^G sites from m7GHub (Song *et al.*, 2020), which contains the human m^7^G sites determined by base-resolution technology (see Table 1), and examined whether WeakRM reported a higher weight near known m^7^G sites.

### 2.2 Weakly supervised learning of RNA modifications

**Fig. 1.**
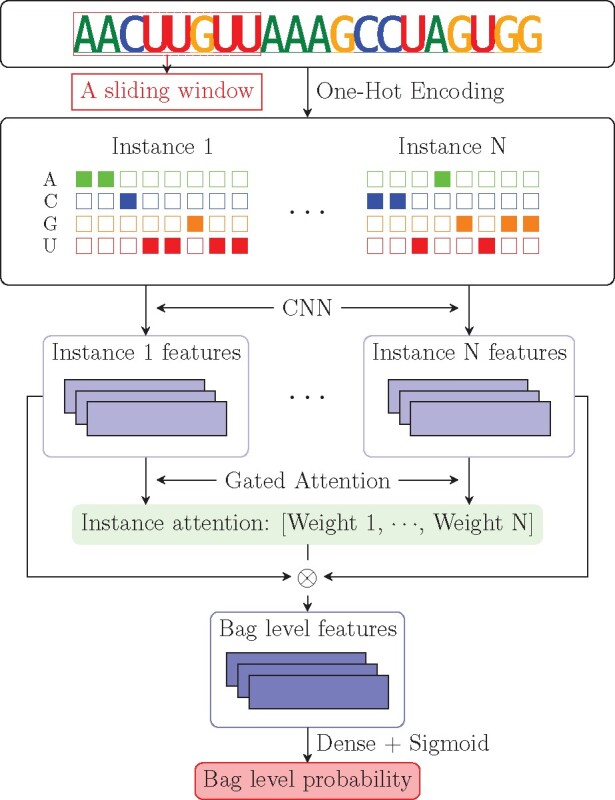
A simplified graphic illustration of the proposed WeakRM framework

We provided in this subsection more details of the proposed WeakRM framework, including data preparation, network architecture, and post-analysis. A simplified illustration of our model is given in Figure 1.

#### 2.2.1 Data preparation

MIL framework treats each RNA sequence as a ‘bag’ with more than one ‘instance’. The target label (with or without RNA modification) is associated with the bag rather than with each instance, indicating whether the RNA modification of interest has occurred within a piece of sequence. In practice, the algorithm divides the entire sequence into multiple overlapping sub-sequences as the ‘instances’ contained within the bag (Zhang *et al.*, 2019, 2020). Specifically, a fixed-length sliding window (length *c*) runs over each bag (length *l*) to capture different portions of it with stride *s*, resulting in total ⌈(l−c)/s⌉+1 instances. Here, the window length *c* and the step-wise shift *s* are two tunable hyperparameters. Reducing *c* and *s* may allow us to locate modifications at a higher resolution but can increase computational load or decrease the prediction accuracy. The difference between those two parameters (*c–s*) reflects the number of nucleotides shared by two adjacent instances.

#### 2.2.2 Data augmentation

**Fig. 2.**
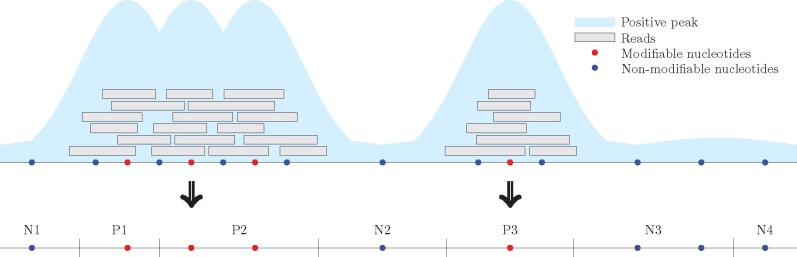
Data augmentation. For FRIP-seq technology, the peaks above 300 nt are formed from multiple sub-peaks corresponding to multiple modifiable nucleotides located in proximity. Therefore, tailoring-wide peaks allow us to obtain multiple sub-sequences, each of which contains at least one site. Two positive sub-sequences P1 and P2 can be generated from a single-wide peak, which corresponds to three RNA modification sites located in proximity. The positive sequence P3 corresponds to a single narrow peak, which may be generated from a single modifiable nucleotide. On the other hand, any sub-sequence from the negative region remains negative, such as N3 and N4

A salient feature of the FRIP-seq protocol, including acRIP-seq, hMeRIP-seq and m^7^G-MeRIP-seq, is that a single modifiable nucleotide, in theory, can only generate a narrow peak (regions enriched with RNA modification signal) of around 100–300 nt long, depending on the peak calling algorithm and the fragment length in the protocol. Very wide peaks, in theory, reflect multiple modifiable nucleotides located in proximity. This property allows us to break up the labeled long sequence into shorter pieces and still ensure a reliable label (see Fig. 2). We used this property for random cropping data augmentation. In each epoch of training, each sequence will be input to the model once. To ensure that the trimmed positive sequences contain at least one RNA modification site, for those peaks with a width greater than 400 nt, different fragments of 3/4 length were randomly selected each time and trained with the same label. Although the actual amount of data has not increased, such random cropping ensures that the same target will not always appear in the same position of the corresponding sequence, which helps our model generalize better.

#### 2.2.3 Model architecture

Starting from the input layer, this sub-section presents a detailed description of the proposed weakly supervised learning framework. The first step in feeding RNA sequences into the WeakRM model is to numerically represent the nucleotides. One-hot encoding is a common way in deep learning-based models which maps each nucleotide into a vector of size 4 (A → [1, 0, 0, 0]^T^, C → [0, 1, 0, 0]^T^, G → [0, 0, 1, 0]^T^ and U → [0, 0, 0, 1]^T^).

To pursue improved resolution in the prediction of RNA modification, instance length is often set to a small value like 50 nt. The used model architecture is as follows: the first convolutional layer captures motifs; a max-pooling layer removes weak features and enlarges the receptive field; a dropout layer prevents overfitting in training, and the second convolutional layer learns local dependencies among motifs. Each instance passes through the same networks (weights are shared) and outputs instance-level features.

#### 2.2.4 Score function

As referred to earlier, in the case of the MIL problem, only an overall binary label associated with the input bag can be assessed. Therefore, how to obtain bag-level probabilities from instance-level features without instance-level labels becomes the key to the MIL framework. Generally, we can divide existing label probability modeling methods into two categories:

1. *The instance score merging approach*: this method requires the construction of an instance-level classifier to estimate the score of each instance. A chosen pooling method then aggregates all instance scores and returns the bag-level class (probability of at least one RNA modification site contained within the input sequence).
2. *The instance feature merging approach*: this method aims at obtaining bag-level feature representations using weighted summation along instance embeddings. The bag-level features are subsequently fed into the final classifier.

Maximum and average are the two most common fusion methods for score merging. However, max-pooling only extracts information concerning the most favored instance, which overlooks other valuable instances and may suffer from outliers. This weakness can even be amplified when our subsequence instances overlap with each other and are therefore highly correlated. Average pooling, on the other hand, assigns equal weights to all instances, which ignores the fact that our instances are sparsely distributed. Other score merging approaches such as, log-sum-exp pooling (Ramón and Raedt, 2000) and noisy-or (Maron and Lozano-Perez, 1997) share a common drawback that they are rule-based and not learnable.

Noisy-and (Kraus *et al.*, 2016) is the fusion method preferred in WSCNN and WSCNNLSTM (Zhang *et al.*, 2019, 2020). Unlike the above methods, it offers a learnable threshold and an auxiliary hyper-parameter for tuning. However, it is still built based on mean scores of instances, which may suffer from the same disadvantage of average pooling.

In our framework, we used the gated attention (Dauphin *et al.*, 2017) as our score function. As a feature merging approach, gated attention uses a three-layer neural network to learn weights *a_k_* of the low-dimensional representation of each instance and obtains the bag-level embedding according to the equation $z=\sum_{k=1}^{K}a_{k}h_{k}$, where $\left\{h_{1},\ldots ,h_{k}\right\}$ is a bag of *K* instance features. When calculating the weights, a gating mechanism (Dauphin *et al.*, 2017) provides a learnable sigmoid non-linearity sigm(·) to enhance the tanh non-linearity tanh(·). A fully connected layer then takes the element-wise multiplication of two non-linearities and returns the gated attention weigths for each instance as presented in equation (1), where w,V and **U** are parameters in layers and ⊤ stands for transpose.

(1) $$a_{k}=\frac{\;\text{exp}\;\{w^{⊤}(\tanh(Vh_{k}^{⊤})⊙\text{sigm}(Uh_{k}^{⊤}))\}}{\sum_{j=1}^{K}\;\text{exp}\;\{w^{⊤}(\tanh(Vh_{j}^{⊤})⊙\text{sigm}(Uh_{j}^{⊤}))\}}$$

Attention measures the degree of similarity among instances and thus is suitable for our context-dependent data. The softmax activation function ensures that all weights add up to 1, which makes the score function invariant to bag size. In addition, the learnable weights indicate the contribution of each instance to bag-level probability. Therefore, the selected method not only effectively leverages all underlying information of instances but also gives an estimation of the site-containing regions. Ideally, the instance that covers a modification should have a specific pattern (motif) and contribute most (highest attention weight) to bag-level prediction.

### 2.3 Validation of site prediction

**Fig. 3.**
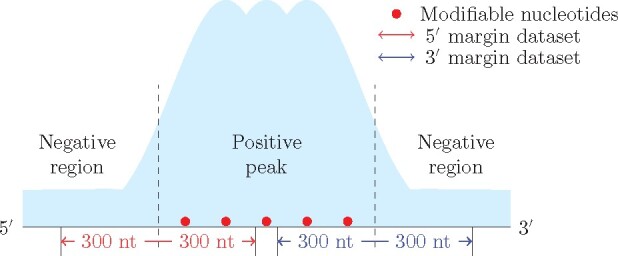
Data for validation of site prediction. A total of 600 nt of sequences were extracted from both the $5^{\prime }$ and $3^{\prime }$ side of the identified peaks, with 300 nt within the peak and 300 nt outside of the peak, respectively. RNA modification sites are expected to appear on the $3^{\prime }$ half for sequences extracted from the $5^{\prime }$ end of the peak or the $5^{\prime }$ half for those from the $3^{\prime }$ end of peaks

Aside from distinguishing the RNA modification-containing and non-containing sequences, a key purpose of our model is to identify the sub-regions containing RNA modifications from a long input sequence. Unfortunately, for hm^5^C and ac^4^C, their transcriptome map of base-resolution is not yet available. Therefore, we developed a validation approach based on low-resolution data. For each peak, we obtained the two marginal areas connecting the negative and positive regions. Specifically, 600 nt of sequences were extracted from both the $5^{\prime }$ and $3^{\prime }$ side of the identified peaks, with 300 nt within the peak and 300 nt outside of the peak, respectively. RNA modification sites are expected to be on the $3^{\prime }$ half for the sequences extracted from the $5^{\prime }$ end of the peak or the $5^{\prime }$ half for those extracted from the $3^{\prime }$ end of the peak (see Fig. 3). We can then check whether this is consistent with the predictions made by WeakRM.

To demonstrate the effectiveness of our model more convincingly, we select m^7^G RNA internal modification data for further validation. Both non-base and single-base techniques are available for m^7^G, which allows us to train our model using peak data and validate using ground truth base-resolution sites. To visually display the results, we placed the known modifiable sites in the center and extracted 300 flanking regions on both sides to obtain a set of 601 nt sequences. For both cases, we picked the most important instance from each true positive bag, recorded their distance to the middle point and plotted their distributions.

### 2.4 Model interpretation

Interpretability of predictive models is often highly desired for biological systems. In the case of RNA modification prediction, this refers to finding the recurring sequence patterns preferred by the model and elucidating the difference between the high-weight and low-weight instances.

Existing motif discovery methods for neural networks can generally be divided into two types. One method is to extract the weights of convolutional kernels in the first network layers, count the occurrence of nucleotides that activate the kernels, and visualize them as position weight matrices (Alipanahi *et al.*, 2015; Kelley *et al.*, 2016). However, these methods only analyze the low-level representation captured by the model, without considering the fact that the neural network learns distributed patterns and makes decisions through the combination of multiple neurons in multiple layers. The other method to interpret predictions is based on the gradients of the output score with respect to the input nucleotide, which follows the natural design of neural networks (back-propagation). The gradient can be analogous to the coefficients in a linear model. Various methods have been developed in the past few years quantifying either the gradient itself (Simonyan *et al.*, 2014) or the products of the gradient and feature values (Bach *et al.*, 2015; Shrikumar *et al.*, 2017; Sundararajan *et al.*, 2017).

In our framework, the integrated gradients (IG) method (Sundararajan *et al.*, 2017) was chosen to quantify the attribution scores of each input feature. As formulated in equation (2), the score sums the gradients of interpolated points along the linear path from the base reference $x^{\prime }$ to the inputs *x*. In practice, trapezoidal IG (Sotoudeh and Thakur, 2019) was used, which is closer to the real theory in the calculation.

(2) $${\text{IG}}_{i}(x)=(x-x^{\prime })\times \sum_{k=1}^{m}\frac{\partial F\left(x^{\prime }+\frac{k}{m}\times (x-x^{\prime })\right)}{\partial x}\times \frac{1}{m}$$

As suggested in Kindermans *et al.* (2019), the reliability of IG depends on the choice of the reference input. Instead of feeding a zero matrix or a fixed letter frequencies matrix into the model as a reference input, we shuffled the original input to construct a reference sequence while retaining the dinucleotide frequencies. This dinucleotide shuffled reference is consistent with the case of regulatory proteins prediction presented in Shrikumar *et al.* (2017) and is suggested in TF-MoDISco (Shrikumar *et al.*, 2018).

Through the visualization of the per-base contribution score generated by the IG method as a saliency map, we were able to identify the portion of each sequence that has a substantial contribution to the prediction. However, there is still a need for systematic analysis to generate a high-quality consensus motif for target modification. TF-MoDISco (Shrikumar *et al.*, 2018), which was developed on the transcription factor, provides a solid solution to generate non-redundant motifs from sequences and the corresponding base-resolution important scores. Segments of the input that are highly relevant to prediction are first identified in all regions of test sequences, and then their contribution scores are clustered and aligned into a motif. This method is applicable to RNA modification analysis from both the biological and computational perspectives, except for the setting of the reverse complementary strands.

## 3 Results and discussion

### 3.1 Model validation on m^7^G data

N7-methylguanine (m^7^G) has traditionally been considered a cap modification of mRNAs. Recent studies identified its widespread internal existence and pivotal roles in translation control (Zhang *et al.*, 2019). Since we previously established benchmark datasets and developed a base-resolution m^7^G predictor (Song *et al.*, 2020), we first verify the proposed WeakRM on m^7^G data. The availability of base-resolution profiling (m^7^G-seq) also enabled a more reliable validation using data produced from an independent biotechnology (Zhang *et al.*, 2019).

To reduce the number of false-positive samples, we extracted the sequences that appear as peaks in both cell lines (HeLa and HepG2) as the positive samples, as in the case of the original study (Zhang *et al.*, 2019). For negative data, only the sequences that appeared as negative in both two cell lines were used. The model performance of each cell line data and two-way cross cell line evaluation were also provided in Supplementary Table S1. We treated the base-resolution m^7^G-seq sites as our ground truth data and extracted the sequences from the 300 nt flanking region on both sides to form a testing sequence of 601 nt. It was expected that the central instance covering the known m^7^G site should have greater attention weights.

#### 3.1.1 Prediction performance

To reduce the potential perturbation of model performance caused by randomness in data splitting, data augmentation, and the scoring function used, an evaluation was performed using 10-fold cross-validation over the low-resolution m^7^G datasets (m^7^G-MeRIP-seq) to produce a reliable comparison. The data were evenly divided into 10 parts, each with the same amount of positive and negative peaks. For all models, an instance length of 50 nt and a stride size of 10 nt were chosen because they generally have better performance on the m7G dataset (see Supplementary Table S2). As shown in Table 2, WeakRM outperformed WSCNN under all three evaluation metrics (especially for the average area under ROC curves measure, 0.896 versus 0.862). Equipped with random cropping data augmentation, an overall improvement can be observed, which indicates that by looking at the different sub-sequences of the input, our model can generalize better. Although the data augmentation does not increase the actual amount of data, random cropping can ensure that the same target does not always appear in the same position in the corresponding sequence. In practice, we have observed that the performance of the WeakRM can be further improved by using the LSTM layer.

**Table 2.** Predictive performance on m^7^G MeRIP data with standard deviation

Model	AUROC	AP	Accuracy
WSCNN (Max.)	0.766(±0.072)	0.762 (±0.044)	0.667(±0.023)
WSCNN (Avg.)	0.664(±0.032)	0.706 (±0.043)	0.628(±0.024)
WSCNN (noisy)	0.775(±0.055)	0.789 (±0.062)	0.705(±0.046)
WSCNNLSTM (Max.)	0.849(±0.013)	0.837 (±0.015)	0.773(±0.015)
WSCNNLSTM (Avg.)	0.851(±0.021)	0.858 (±0.022)	0.760(±0.024)
WSCNNLSTM (noisy)	0.862(±0.021)	0.870 (±0.019)	0.772(±0.023)
**WeakRM**	0.892(±0.014)	0.889 (±0.020)	0.815(±0.017)
**WeakRM (crop)**	**0.896 (±0.013)**	**0.897 (±0.016)**	**0.816 (±0.015)**

*Note*: All methods were evaluated using the same datasets.

#### 3.1.2 Location estimation

**Fig. 4.**
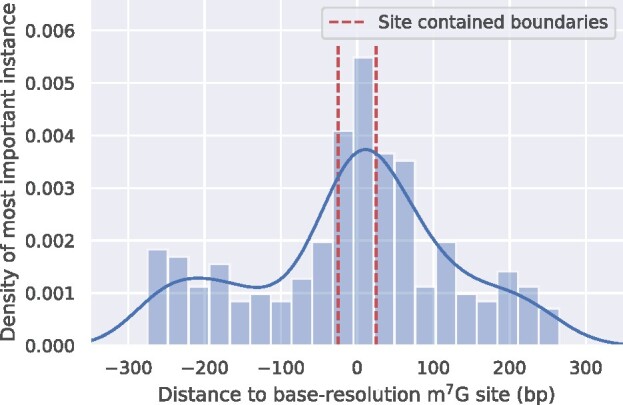
Density of highest weight instance location on single-base m^7^G validation data. The known site is placed in the center (0 on *X*-axis). The red dashed line indicates the boundaries of instances that contain the m^7^G site

To explore the potential of identifying RNA modification sites from the instances with high attention weights, we applied the well-trained model built on low-resolution m^7^G-MeRIP-seq data to the sequences generated from base-resolution data, for which we know the exact location of m^7^G sites. For each predicted true positive sequence, we selected the most important instance based on the gated attention weight and visualized their relative distances to the known m^7^G sites in the middle. As shown in Figure 4, a strong peak of the distribution appeared near the location of the known m^7^G site (0 on *X*-axis). Given the instance length (50) and stride (10), there exist five instances containing the m^7^G site detected using base-resolution technology in each bag. The instances with the m^7^G site near their centers are likely to produce the highest attention weights. There are still some high-weight instances that do not contain known m^7^G sites. Among them, those near the center area showed a higher probability to be high-weight instances. This may indicate that WeakRM has captured some sequence patterns that are not immediately close to the m^7^G site, or there existed previously undetected m^7^G sites and the modification exhibits a clustering effect, as previously observed in the case of N6-methyladenosine (m^6^A) (Chen *et al.*, 2019). Our results provided strong evidence that the proposed WeakRM framework has the potential to estimate the location of RNA modifications from low-resolution data alone.

#### 3.1.3 Model interpretation

To gain further insights into the sequence-dependent forming mechanism of RNA modification unveiled by our proposed WeakRM, we implemented a trapezoidal IG method with zero matrixes, fixed letter frequencies (GC content) and dinucleotide shuffled references to obtain attribution maps on the input instances. For each test sequence, we simulated 50 shuffled references and used 20 steps in calculating IG values. As our input data were all one-hot encoded, such scores of contribution can be easily transformed to the importance of each nucleotide.

**Fig. 5.**
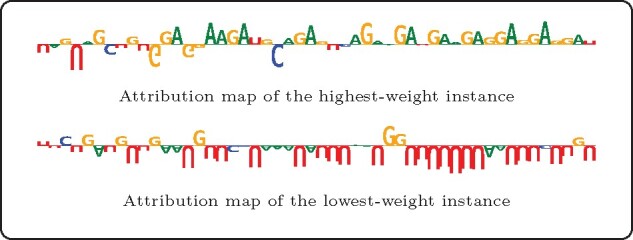
Attribution maps for the instance with the highest and lowest attention weight from the single-base m^7^G sequence with highest predicted probability

As shown in Figure 5, our proposed model assigned high attention weights to adenine (A) and guanine (G) enriched areas, which coincides with the two major bases in known motifs. Our target, m^7^G, is a modification that happens on nucleotide G. By observing the distribution of the G contribution score, we observe that not all guanines have received high values, and some may even have a negative impact on the positive peak prediction. Therefore, such interpretable scores can be a potential way to further narrow the range of predicted modified sites.

On the other hand, continuous cytosine (C) and uracil (U) patterns were abundant in the low weight instances (Fig. 5), which often made a negative contribution to the prediction of m^7^G-containing region. For visualization purposes, we individually normalized the contribution scores for each instance, which resulted in comparable score ranges between the high-weight and low-weight instances. However, the former is usually much larger than the latter in the absolute sense.

**Fig. 6.**
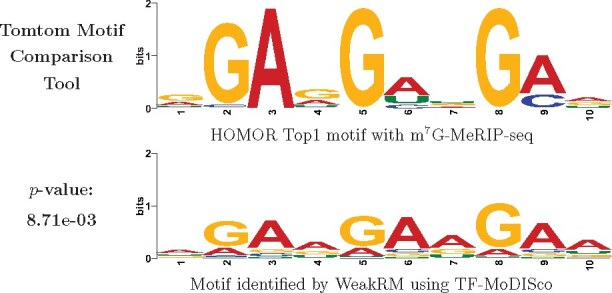
Match motif identified by WeakRM to Top-1 motif identified with m^7^G-MeRIP-seq by HOMER software

Attribution maps show only the model preference of nucleotides in every single test. To summarize the recurring motifs captured by WeakRM, the current general pipeline carries out high-weight *k*-mers selection, clustering of similar patterns, and multiple sequence alignment for obtained motifs in each cluster. In our work, we use TF-MoDISco to extract consensus motif from instances with higher than average weights. A great advantage of TF-MoDISco is that it provides the continuous Jaccard similarity calculation to carry out alignment directly based on contribution scores instead of only selecting the most important bases. By allowing three gaps and two mismatches and trimming using overall letter frequencies, we found one consensus motif, given in the Figure 6. Compared with the known motifs identified with m^7^G-MeRIP-seq using HOMER software and reported in data source paper (Zhang *et al.*, 2019), we found the motif learned by WeakRM can be matched to the Top-1 known motif with a *P*-value of 8.71e-03. The *P*-value here represents the probability that a random motif of the same width has the same or better matching score as the target. The value 8.71e-03 is sufficient to infer a high similarity between the motifs.

### 3.2 Case study 1: prediction of 5-hydroxymethylcytidine

Recent studies have revealed a relatively high abundance of 5-hydroxymethylcytidine (hm^5^C) in fly and mouse brains (Delatte *et al.*, 2016; Miao *et al.*, 2016). To date, high-throughput profiling of hm^5^C is only possible via the hMeRIP-seq technique (Delatte *et al.*, 2016), which reports regions enriched with hm^5^C signal (or low-resolution hm^5^C-containing peaks). Due to the existence of multiple Cytosines within such regions, it is not exactly clear which specific Cytosine can be modified by hydroxylmethylation. When WeakRM was applied to hm^5^C, all sequences under the hm5C peaks from *Drosophila* S2 cells hMeRIP-seq data were used as the positive samples. Negative samples were collected from negative regions that do not intersect with the positive peaks in the same cell line. These samples were further selected and randomly trimmed to fit the number and width of the positive sequences.

#### 3.2.1 Weakrm outperformed competing algorithms

We randomly split the dataset into training, validation and testing sets using a ratio of 8:1:1. Each dataset contains an equal number of positive and negative sequences and of roughly the same size distribution. The area under the ROC curve (AUROC), average precision (AP) and accuracy with 0.5 threshold were selected as the main evaluation metrics during performance evaluation.

As shown in Table 3, the proposed WeakRM model achieved the best performance with respect to all three evaluation metrics compared with WSCNNLSTM with Noisy-and fusion method. Random cropping data augmentation effectively improved the predictive performance of WeakRM on hm^5^C data, and the improvement is more obvious than that of m^7^G. This may be because the peak of hm^5^C has a larger width overall. Furthermore, through data augmentation, the training is more stable.

**Table 3.** Predictive performance on hm^5^C data with standard deviation

Model	AUROC	AP	Accuracy
WSCNNLSTM	0.889 (±0.007)	0.883 (±0.011)	0.775 (±0.022)
**WeakRM**	0.894 (±0.014)	0.907 (±0.007)	0.792 (±0.025)
**WeakRM (crop)**	**0.909 (±0.003)**	**0.912 (±0.003)**	**0.823 (±0.018)**

*Note*: All methods were evaluated using the same datasets.

The bold indicates the proposed method and the best performance under different evaluation metrics.

It is worth noting that, to the best of our knowledge, there exist only two computational approaches iRNA5hmC (Liu *et al.*, 2020) and iRNA5hmC-PS (Ahmed *et al.*, 2020) for predicting hm^5^C RNA modification from RNA sequences. Both methods were based on strongly supervised learning of the same dataset (Delatte *et al.*, 2016) as the one used in our study. Although both of them achieved positive predicting results (AUROC of 0.70 and 0.86), the performance of WeakRM is even better (AUROC of 0.909), suggesting the advantage of the proposed computational framework.

#### 3.2.2 Weakrm detected sub-regions containing hm^5^C

**Fig. 7.**
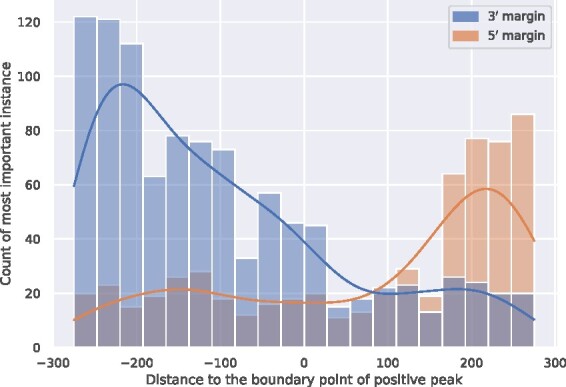
Location of the most important instances reported on the peak-margin datasets. A total of 600 nt of sequences were extracted from both the $5^{\prime }$ and $3^{\prime }$ margins of the called peaks, with 300 nt within the peak and 300 nt outside of the peak, respectively. With this setting, hm^5^C sites are expected to appear on the $3^{\prime }$ half for sequences extracted from the $5^{\prime }$ end of the peak or the $5^{\prime }$ half for the sequences extracted from the $3^{\prime }$ end of peaks. It is worth noting that, a single hm^5^C site can generate a peak of more than 200 bp in the MeRIP-seq data, so the true location of the hm^5^C site should not be immediately before or after the edges of the peaks (coordinate 0 of *X*-axis). These are all consistent with the distribution pattern of the most important instances of the sequences

A potentially useful application of the trained WeakRM model is to detect the sub-regions containing hm^5^C out of a long input sequence. Since there exist no high-throughput approaches for profiling hm^5^C at base resolution, ground truth data (base-resolution hm^5^C sites) was unavailable. As such, we developed a new peak margin-based method to demonstrate the effectiveness of our model in identifying the sub-regions containing hm^5^C out of a long input sequence. Specifically, the peaks from hMeRIP-seq data naturally have two margins, one toward the $5^{\prime }$ end and the other towards the $3^{\prime }$ end, which allows us to construct datasets containing both positive and negative sequences. Ideally, our model should assign higher weights to the positive side while giving lower weights to the negative. To display our results, for each sequence, we selected the most important instance and recorded its relative position to the margin (0 on axis, which indicates the margins of the peaks). We show in Figure 7 the density of these relative distances. It is clear that the most important instances were enriched on the positive sides of the corresponding datasets, which provided strong evidence that our model can discriminate hm^5^C-containing sub-regions against the rest of the sequences.

### 3.3 Case study 2: prediction of N4-acetylcytidine

Recently, despite some controversy (Sas-Chen *et al.*, 2020), N4-acetylcytidine (ac^4^C) were identified on poly(A) RNA isolated from a variety of human cells (Dong *et al.*, 2016; Guo *et al.*, 2020). Similar to hm^5^C, high-throughput profiling approaches of base resolution is not yet available for ac^4^C. Only low-resolution epitranscriptome data are available for this modification via acRIP-seq, making it a suitable subject for weakly supervised learning. When preparing the data, we extracted the ac^4^C peaks mutually detected in cell lines as the positive samples. Correspondingly, the negative data were extracted from the intersection of negative regions as well. The negative samples were further selected to match the number and width distribution of the positive samples.

#### 3.3.1 Weakrm outperformed competing algorithms

To the best of our knowledge, only two computation methods, PACES (Zhao *et al.*, 2019) and XG-ac4C (Alam *et al.*, 2020), have been developed so far for the prediction of ac^4^C from sequences, and again, both were based on strong supervision. Although both approaches achieved positive prediction performance, they require a very specific sequence pattern and consider only sequences that have at least five continuous CXX repeats, which may limit the applicable scope of these methods. Compared to them, our WeakRM model does not presume any motifs of ac^4^C in advance and lets the neural networks learn the sequence patterns associated with ac^4^C directly from the complete low-resolution epitranscriptome data, and is thus applicable to all input sequences with no prerequisites.

Since the webserver of XG-ac4C allows a fixed input of length 415 nt, we constructed our ac^4^C dataset by selecting the peaks that do not exceed 415 nt and resizing the width of selected peaks to 415 nt for a fair comparison. Following the setting in our previous example, we used the same ratio 8:1:1 to split the dataset into the training, validation, and testing sets. Each dataset contains an equal number of positive and negative samples.

As shown in Table 4, although trained by strong supervision, XG-ac4C still achieved positive results on our newly constructed dataset. Our WeakRM approach performed the best and is more robust than WSCNNLSTM. Nevertheless, it may not be appropriate to compare directly WeakRM with existing methods based on strong supervision, as they have different assumptions and goals, and require different experimental settings.

**Table 4.** Predictive performance on ac^4^C data with standard deviation

Model	AUROC	AP	Accuracy
XG-ac4C	0.786	0.774	0.680
WSCNNLSTM	0.912(±0.012)	0.895(±0.014)	0.835(±0.015)
**WeakRM**	0.935(±0.007)	0.925(±0.008)	0.863(±0.009)

*Note*: All methods were evaluated using the same datasets.

The bold indicates the proposed method and the best performance under different evaluation metrics.

#### 3.3.2 Weakrm detected sub-regions containing ac^4^C

**Fig. 8.**
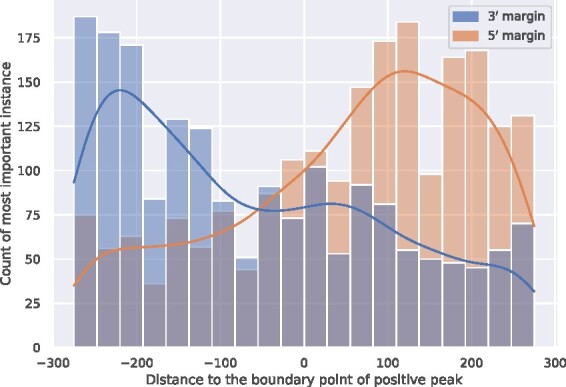
Density plot of the most important instance locations on ac^4^C peak margin datasets. A total of 600 nt of sequences were extracted from both the $5^{\prime }$ and $3^{\prime }$ margins of the ac^4^C peaks, with 300 nt within the peak (positive side) and 300 nt outside of the peak (negative side), respectively. With this setting, the ac^4^C sites are expected to appear on the $3^{\prime }$ half for sequences extracted from the $5^{\prime }$ end of the peak or the $5^{\prime }$ half for sequences from the $3^{\prime }$ end of peaks. It is worth noting that, a single ac^4^C site can generate a peak of more than 200 nt in the MeRIP-seq data, so the true location of hm^5^C site should not appear immediately before or after the edges of the peaks (coordinate 0 of *X*-axis), but with some shifts. These are all consistent with the observed distribution pattern of the most important instances of the sequences.

To demonstrate the ability of the proposed WeakRM algorithm in detecting sub-regions containing ac^4^C out of a long input sequence, we performed the peak margin-based testing as described in Case Study 1. As shown in Figure 8, the high-weight instances were again enriched on the positive sides of the corresponding datasets, showing strong evidence that our model is capable of discriminating ac^4^C-containing sub-regions from the rest of the sequences. Interestingly, compared with the observed patterns for hm^5^C (see Fig. 7), the most important instances of ac^4^C appear closer to the boundary (coordinate 0 of *X*-axis), indicating a better spatial accuracy in detecting RNA-modification containing sub-regions. It may be because compared with hm^5^C, the peaks generated from ac^4^C sites are narrower and have more accurate boundaries. This may be related to the experiment protocol (e.g. fragment size) and peak calling algorithms (e.g. sliding window size) used in their original studies.

## 4 Conclusion

Existing computational approaches for decoding the RNA modifications are mostly based on strong supervision and ideally require epitranscriptome data of base-resolution. Due to technical limitations, such data may not be available for some modifications, such as ac^4^C and hm^5^C. We proposed here the first weakly supervised learning framework WeakRM for learning RNA modifications from low-resolution epitranscriptome datasets, such as those generated from hMeRIP-seq and acRIP-seq.

We validated the proposed WeakRM method on three independent datasets, corresponding to three different RNA modification types (m^7^G, hm^5^C and ac^4^C) and their respective sequencing technologies (m^7^G-MeRIP-seq, hMeRIP-seq and acRIP-seq). We demonstrated that WeakRM substantially improved the prediction performance and applicable scope compared with existing approaches that were based on strong supervision. Importantly, our model captured sequence patterns that are consistent to the known motif detected by HOMOR software, and can vaguely identify regions containing the RNA modifications of interest. These results together demonstrated the generality and effectiveness of our approach for learning from low-resolution epitranscriptome data.

Notably, WeakRM also outperformed the existing weakly supervised learning algorithms for sequence analysis developed on TFBS prediction. This was made possible by two major improvements from the algorithm perspective. First, instead of using the widespread instance score merging approach, we applied an attention-based feature merging strategy to obtain learnable weights for each instance. Second, data augmentation was performed by taking advantage of the salient features of the FRIP-seq protocol via random cropping, which extended the diversity of training samples.

Given the positive results reported in our study, WeakRM and weakly supervised learning framework should make a powerful tool for studying RNA modifications when only low-resolution epitranscriptome data are available.

## Funding

This work was supported by the National Natural Science Foundation of China [31671373]; XJTLU Key Program Special Fund [KSF-T-01]. This work was partially supported by the AI University Research Centre (AI-URC) through XJTLU Key Programme Special Fund (KSF-P-02).

*Conflict of interest*: none declared.

## Supplementary Material

btab278_Supplemenatry_Data
