# Supplementary material for: Weakly supervised learning of RNA modifications from low-resolution epitranscriptome data
Source: Bioinformatics. 2021 Jul 12;37(Suppl 1):i222–30. doi: 10.1093/bioinformatics/btab278 (PMC8336446; doi:10.1093/bioinformatics/btab278)
Supplement: btab278_Supplemenatry_Data [file btab278_supplemenatry_data.pdf]

Table 1 Predictive performance between m^{7}G cell lines

Training	Test	Accuracy	Recall	Precision	F1	MCC	AUROC	AP
HeLa	HeLa	0.8048	0.8290	0.7908	0.8094	0.6104	0.8785	0.8828
HeLa	HepG2	0.7651	0.7358	0.7816	0.7580	0.5311	0.8465	0.8447
HepG2	HepG2	0.805	0.7626	0.8333	0.7964	0.6123	0.8853	0.8846
HepG2	HeLa	0.7387	0.6242	0.8097	0.7049	0.4905	0.8295	0.8281

* For each cell line, we randomly split the dataset into training, validation, and testing set using a ratio of 8:1:1. When the model trained on the HeLa data set is applied to the HepG2 data set, all peaks that do not overlap with the training peaks used in the HeLa model are selected from the HepG2 data set to form a balanced test data set, vice versa.

Table 2 Predictive performance of different instance length and stride

	HeLa		HepG2	
	AUROC	AP	AUROC	AP
Len 40; Stride 5	0.8737	0.8654	0.8842	0.8798
Len 40; Stride 10	0.8695	0.8609	0.8803	0.8825
Len 50; Stride 5	0.864	0.862	0.8847	**0.885**
Len 50; Stride 10	**0.8785**	**0.8728**	**0.8853**	0.8846

*For each cell line, all evaluations were done using the same datasets.

Table 3 Predictive performance on base-resolution m^{7}G data using WeakRM with MeRIP-seq data

Accuracy	Recall	Precision	F1	MCC	AUROC	AP
0.6133	0.4259	0.6813	0.5241	0.2445	0.6614	0.6899

*The test dataset consists of 801 positive sequences of 101nt centered at base-resolution m7G sites. The negative sequences were sampled from the site-containing genes, centering at Guanine. Negative data has the same sample size and width as positive data.

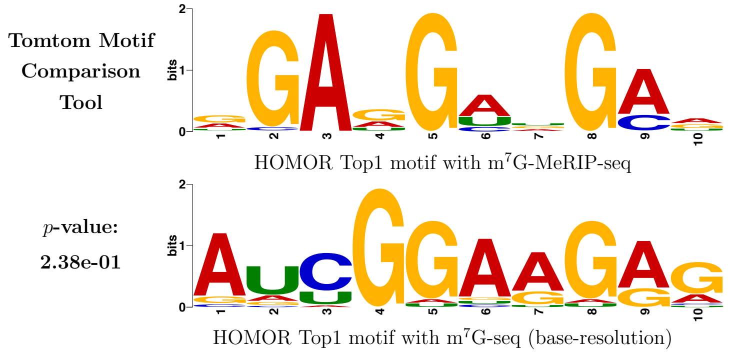

Fig 1. Motif comparison between m^{7}G-seq and m^{7}G-MeRIP-seq. Motifs were obtained using HOMOR. Tomtom motif comparison tool was used to calculate a p-value. The matching result shows that the sequence pattern between the m^{7}G-MeRIP-seq data and the m^{7}G-seq data is slightly different. This may be a reason that WeakRM has limited effectiveness in predicting base-resolution data.
